# Hospitalisation patterns in interstitial lung diseases: data from the EXCITING-ILD registry

**DOI:** 10.1186/s12931-023-02588-y

**Published:** 2024-01-04

**Authors:** Katharina Buschulte, Hans-Joachim Kabitz, Lars Hagmeyer, Peter Hammerl, Albert Esselmann, Conrad Wiederhold, Dirk Skowasch, Christoph Stolpe, Marcus Joest, Stefan Veitshans, Marc Höffgen, Phillen Maqhuzu, Larissa Schwarzkopf, Andreas Hellmann, Michael Pfeifer, Jürgen Behr, Rainer Karpavicius, Andreas Günther, Markus Polke, Philipp Höger, Vivien Somogyi, Christoph Lederer, Philipp Markart, Michael Kreuter

**Affiliations:** 1 Center for Interstitial and Rare Lung Diseases, Thoraxklinik, University of Heidelberg, German Center for Lung Research (DZL), Heidelberg, Germany; 2 Medical Clinic II, Pneumology and Intensive Care Medicine, Klinikum Konstanz, Konstanz, Germany; 3 Hospital Bethanien Solingen, Clinic of Pneumology and Allergology, Center of Sleep Medicine and Respiratory Care, Solingen, Germany; 4 Chest Clinic Immenhausen, Immenhausen, Germany; 5 Outpatient Center for Pulmonology, Warendorf, Germany; 6 Outpatient Center for Pulmonology, Fulda, Germany; 7 Department of Medicine II, University Hospital Bonn, Bonn, Germany; 8 Outpatient Center for Pulmonology, Ibbenbüren, Germany; 9 Outpatient Center for Pulmonology and Allergology, Bonn, Germany; 10 Outpatient Center for Pulmonology, Böblingen, Germany; 11 Outpatient Center for Pulmonology, Rheine, Germany; 12 Institute of Health Economics and Healthcare Management, Helmholtz Centre Munich GmbH, German Research Centre for Environmental Health, German Centre for Lung Research (DZL), Comprehensive Pneumology Centre Munich (CPCM), Neuherberg, Germany; 13 IFT Institut für Therapieforschung, Centre for Mental Health and Addiction Research, Munich, Germany; 14 Outpatient Center for Pulmonology, Augsburg, Germany; 15 Medical Clinic II, University of Regensburg and Klinikum Donaustauf, Donaustauf, Germany; 16 Department of Medicine V, LMU University Hospital, LMU Munich, Comprehensive Pneumology Centre, German Center for Lung Research (DZL), Munich, Germany; 17 Patient Support Group Lungenfibrose e.V., Essen, Germany; 18 Medical Clinic II, University Hospital Giessen, Universities of Giessen and Marburg Lung Centre (UGMLC), German Center for Lung Research (DZL), Giessen, Germany; 19 Mainz Center for Pulmonary Medicine, Departments of Pneumology, ZfT, Mainz University Medical Center and of Pulmonary, Critical Care & Sleep Medicine, Marienhaus Clinic Mainz, Mainz, Germany; 20 Medical Clinic V (Pneumology), Cardiothoracic Centre, Campus Fulda, University Medicine Marburg, Fulda, Germany

**Keywords:** ILD, IPF, Hospitalisation, Prognosis, Risk factors

## Abstract

**Background:**

Interstitial lung diseases (ILD) comprise a heterogeneous group of mainly chronic lung diseases with more than 200 entities and relevant differences in disease course and prognosis. Little data is available on hospitalisation patterns in ILD.

**Methods:**

The EXCITING-ILD (Exploring Clinical and Epidemiological Characteristics of Interstitial Lung Diseases) registry was analysed for hospitalisations. Reasons for hospitalisation were classified as all cause, ILD-related and respiratory hospitalisations, and patients were analysed for frequency of hospitalisations, time to first non-elective hospitalisation, mortality and progression-free survival. Additionally, the risk for hospitalisation according to GAP index and ILD subtype was calculated by Cox proportional-hazard models as well as influencing factors on prediction of hospitalisation by logistic regression with forward selection.

**Results:**

In total, 601 patients were included. 1210 hospitalisations were recorded during the 6 months prior to registry inclusion until the last study visit. 800 (66.1%) were ILD-related, 59.3% of admissions were registered in the first year after inclusion. Mortality was associated with all cause, ILD-related and respiratory-related hospitalisation. Risk factors for hospitalisation were advanced disease (GAP Index stages II and III) and CTD (connective tissue disease)-ILDs. All cause hospitalisations were associated with pulmonary hypertension (OR 2.53, p = 0.005). ILD-related hospitalisations were associated with unclassifiable ILD and concomitant emphysema (OR = 2.133, p = 0.001) as well as with other granulomatous ILDs and a positive smoking status (OR = 3.082, p = 0.005).

**Conclusion:**

Our results represent a crucial contribution in understanding predisposing factors for hospitalisation in ILD and its major impact on mortality. Further studies to characterize the most vulnerable patient group as well as approaches to prevent hospitalisations are warranted.

**Supplementary Information:**

The online version contains supplementary material available at 10.1186/s12931-023-02588-y.

## Background

Interstitial lung diseases (ILD) comprise a heterogeneous group of more than 200 mainly chronic diseases [1–3]. The diagnosis of an ILD is made in a multidisciplinary context considering clinical, radiological, and pathological aspects [2, 3]. With the advent of new therapeutic options including antifibrotic therapies for pulmonary fibrosis [4] or new approaches in inflammatory diseases such as systemic sclerosis [5], ILD-associated morbidity and mortality have reduced, but are still relevant. A recent global burden of disease study describes that incidence, mortality, and disability-adjusted life-years of different ILDs are increasing [6]. Furthermore, ILDs play a decisive role regarding the economic burden on healthcare systems, mainly due to costs associated with the pharmacological management and due to hospitalisations [7]. Hospitalisations have also a great impact on quality of life and prognosis, mainly hospitalisations for respiratory reasons [8]. These include e.g. pulmonary infections, acute exacerbations, or ILD related spontaneous pneumothoraces. A large study with almost 600 patients suffering from idiopathic pulmonary fibrosis (IPF) reported that all cause and respiratory-related hospitalisations were strongly associated with mortality [8]. Especially, acute exacerbations have a poor outcome in many ILDs as shown for IPF [9], hypersensitivity pneumonitis (HP) [10] and rheumatoid arthritis (RA) [11], as well as for progressive fibrosing ILDs [12]. Risk factors for hospitalisations are mainly unknown, but one study suggested that different severities of exacerbations exist [9]. In addition, an association between lung functional decline and an increased risk of hospitalisation in systemic sclerosis-associated interstitial lung disease (SSc-ILD) has recently been reported [13] and according to Cottin et al. also comorbidities, mainly cardiovascular events, are associated with frequent hospitalisations [14].

Important insights on these aspects could be obtained through registries. While several registries collecting data on IPF are available, e.g. the German INSIGHTS IPF registry, only few registries including all different ILD subtypes are existing worldwide [15]. The “Exploring Clinical and Epidemiological Characteristics of Interstitial Lung Diseases” (EXCITING-ILD) registry, a multicentre noninterventional, prospective, and observational disease and outcomes registry conducted by the German Center for Lung Research aimed to assess outcomes across all ILD subtypes [16]. Aim of the current work was to assess hospitalisations in different ILD subtypes, impact on mortality and influencing factors, e.g. comorbidities.

## Methods

### Study design

The EXCITING-ILD registry provides sociodemographic and medical data on ILDs in Germany from different healthcare facilities including ambulatory, in-patient, scientific pulmonology organisations, as well as patient support groups [16]. The study was approved by the Ethics Committee of the Medical Faculty of the University of Heidelberg, Germany (S-525/2013) as well as by all local ethics committees of the participating centres.

The registry protocol has been published elsewhere [16]. Shortly, incident and prevalent patients with any ILD were included and followed prospectively for at least 36 months and a maximum of five years. All patients with a minimum of one documented post-baseline visit were entered into the full analysis set (FAS) [16]. Data collected included baseline and follow up variables. Furthermore, demographic data, ILD subtypes, diagnostic procedures, distinct comorbidities, ILD management, and outcomes including data on hospitalisation and associated factors were assessed [16].

### Statistical analysis

For our analyses, the following definitions applied: All Hospitalisations were further divided into ILD-related and ILD-unrelated. ILD-related hospitalisations were caused by or associated with ILD including hospitalisations for elective procedures. For further analyses (time-to-event endpoints) only non-elective ILD-hospitalisation from date of inclusion were considered. A respiratory hospitalisation was defined as exclusively non-elective caused by pneumonia, ILD exacerbation or pneumothorax. Multiple entries for the reason of admission were possible, which means that one hospitalisation can be associated with several of the listed reasons (e.g. pneumonia and ILD exacerbation). For time to first hospitalisation only non-elective hospitalisations were considered. In particular, time to first non-elective hospitalisation was defined as the first non-elective hospitalisation from inclusion to the registry. Hospitalisations were included into the following definitions of progression free survival (PFS): (A) ΔForced vital capacity (FVC) ≥ 10% or respiratory hospitalisation or death *and* (B) ΔFVC ≥ 10% or all cause hospitalisation or death. The GAP index, in the present study considered as a possible risk factor for hospitalisations, is a point scoring stage model based on clinical and physiologic variables to predict mortality in patients with pulmonary fibrosis. Higher GAP scores indicate worse health state [17].

Observational data were analysed descriptively using means with standard deviations (SD) and percentages. For time-to-event endpoints (hospitalisations, mortality, PFS), Kaplan–Meier curves were used to estimate event-free survival times. Confidence intervals were set with a two-sided level of 95% [16]. For the comparison of two or more Kaplan–Meier curves a log-rank test was performed. To quantify the difference between groups, hazard ratios and corresponding two-sided 95% confidence intervals were estimated based on a Cox proportional hazards model. The p-value of the corresponding Wald-test was calculated. Logistic regression was used to develop a model to predict hospitalisation and to identify variables with a significant influence on hospitalisation. The model was generated using forward selection. By this, the only essential predictors were selected to explain the target variables hospitalisation for any reason, due to ILD and due to respiratory causes. Sex, age, body mass index (BMI), pulmonary function parameters, ILD subtype, pulmonary hypertension, reflux, concomitant emphysema, and smoking behaviour were considered as possible predictors. First, univariable regression models were set up to predict hospitalisation (5% significance level) and second multiple regression models were generated. Data analysis was performed using the statistics software R (R version 4.1.2).

## Results

### Study population

In total, 601 patients were included in 31 centres. 60.7% were male and the mean age was 64.3 years (Table 1). All different ILD subtypes were included: 26.6% sarcoidosis, 25.3% IPF, 9.7% HP, 7.2% CTD- and RA-ILD, 7% NSIP, 5.7% unclassifiable ILD (uILD), 4.2% cryptogenic organizing pneumonia (COP), 2.7% drug-related ILDs (DI-ILD), 2.3% fibrosis in emphysema patients without signs of other ILDS (CPFE), 1.8% pneumoconiosis, 1.2% pulmonary lymphangioleiomyomatosis (LAM), 1.0% eosinophilic pneumonia, 1.0% radiotherapy associated-ILD (RTX-ILD), 0.8% other granulomatous lung disease (other GRAN-ILD), 0.8% desquamative interstitial pneumonia (DIP), 0.7% respiratory bronchiolitis-associated interstitial lung disease (RB-ILD), 0.5% pulmonary alveolar proteinosis (PAP), 0.3% pulmonary Langerhans´ cell histiocytosis (PLCH), and 1.2% others. Mean FVC was 76.4% predicted and most patients were in GAP stage I (Table 1).

**Table 1:** Baseline characteristics of the full analysis set

Characteristics	Total (n = 601)
Male Sex, n (%)	365 (60.7)
Age [years], mean (SD)	64.3 (14.2)
BMI, mean (SD)	27.3 (4.84) n = 588
Familial ILD, n (%)	22 (3.7)
Current or Ex-smoker, n (%)	322 (53.6)
FVC [% predicted], mean (SD)	76.4 (20.8) n = 349
FEV1 [% predicted], mean (SD)	79.2 (20.0) n = 458
FEV1/FVC [% predicted], mean (SD)	105 (13.5) n = 347
DLCo-SB [% predicted], mean (SD)	54.1 (21.6) n = 329
Walking distance in 6-min walk [m], mean (SD)	371 (120) n = 149
GAP-Index, n (%) I II III Missing	275 (45.8)
	164 (27.3)
	128 (21.3)
	34 (5.7)

The total numbers for which the respective values were available are indicated within the rows. Where nothing is indicated, the numbers refer to the total of n=601. *SD* standard deviation, *BMI* body mass index, *ILD* interstitial lung disease, *FVC* forced vital capacity, *FEV1* forced expiratory volume in 1 second, *DLCO* diffusing capacity for carbon monoxide (CO)

### Characterisation of hospitalisations

During a median follow-up of 3 years, 1210 hospitalisations were recorded during the 6 months prior to registry inclusion until the last study visit. 66.1% of these hospitalisations were ILD-related. ILD-related admissions mainly included elective diagnostic procedures (n = 293, 33.5%) and acute exacerbations (n = 182, 20.8%). Other reasons were pulmonary infections (n = 165, 18.9%), initiation of non-invasive ventilation (n = 30, 3.4%), lung cancer or other malignancies (n = 18, 2.1%), pulmonary embolism (n = 14, 1.6%), and pneumothorax (n = 5, 0.6%) (Table 2)*.*

**Table 2:** Reasons for ILD-related admissions

Reason for ILD-related admissions	N = 874 (%)
Pneumonia / pulmonary infection	165 (18.9)
ILD Exacerbation	182 (20.8)
Pneumothorax	5 (0.6)
Suspicion on/diagnosis of lung cancer or other ILD-related malignancies	18 (2.1)
Pulmonary embolism	14 (1.6)
Elective for diagnostic procedure	293 (33.5)
Initiation of NIV	30 (3.4)
Others	167 (19.1)

This table lists all reasons for ILD-related admission. For one hospitalisation several reasons for admission could be considered. ILD= interstitial lung disease, *NIV* non-invasive ventilation

**Fig. 1:**
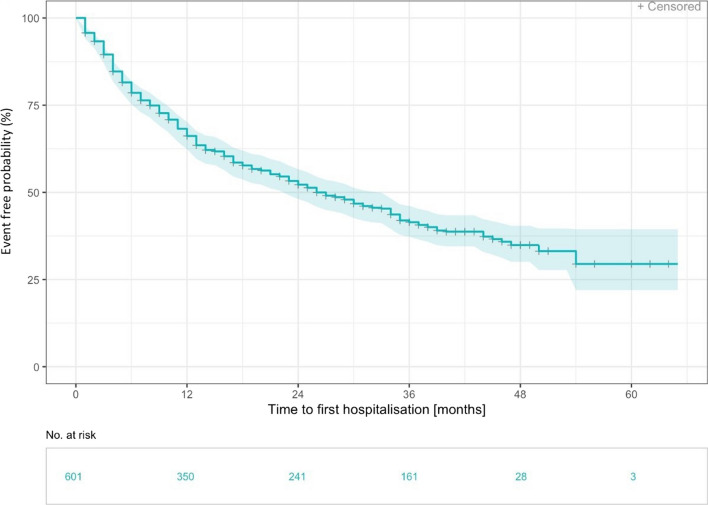
Time to first non-elective hospitalisation in months—Kaplan Meier curve. Time to first non-elective hospitalisation was defined as the first non-elective hospitalisation from inclusion to the registry (n= 322). Median time to first hospitalisation was 26 months

Non-elective hospitalisations (n = 322) occurred most frequently with 59.3% (n = 191) in the 1st year after inclusion into the registry, while in the second year 21.4% of hospitalisations (n = 69) were recorded. 208 events of first hospitalisation due to ILD (34.6%) were reported. Median time to first hospitalisation was 50.8 months (range 0.1 to 50.8 months, Additional file 1: Fig. S1) compared to 26 months (range 0,1 to 50.8 months) in all cause hospitalisations (Fig. 1).

### Progression free survival and mortality

Median progression free survival time was 25.7 months (range 0.1 to 59.63 months) for PFS definition A (i.d. ΔFVC ≥ 10% or *respiratory* hospitalisation or death) and 13.9 months (range 0.1 to 59.63 months) for PFS definition B (ΔFVC ≥ 10% or *all cause* hospitalisation or death).

**Fig. 2:**
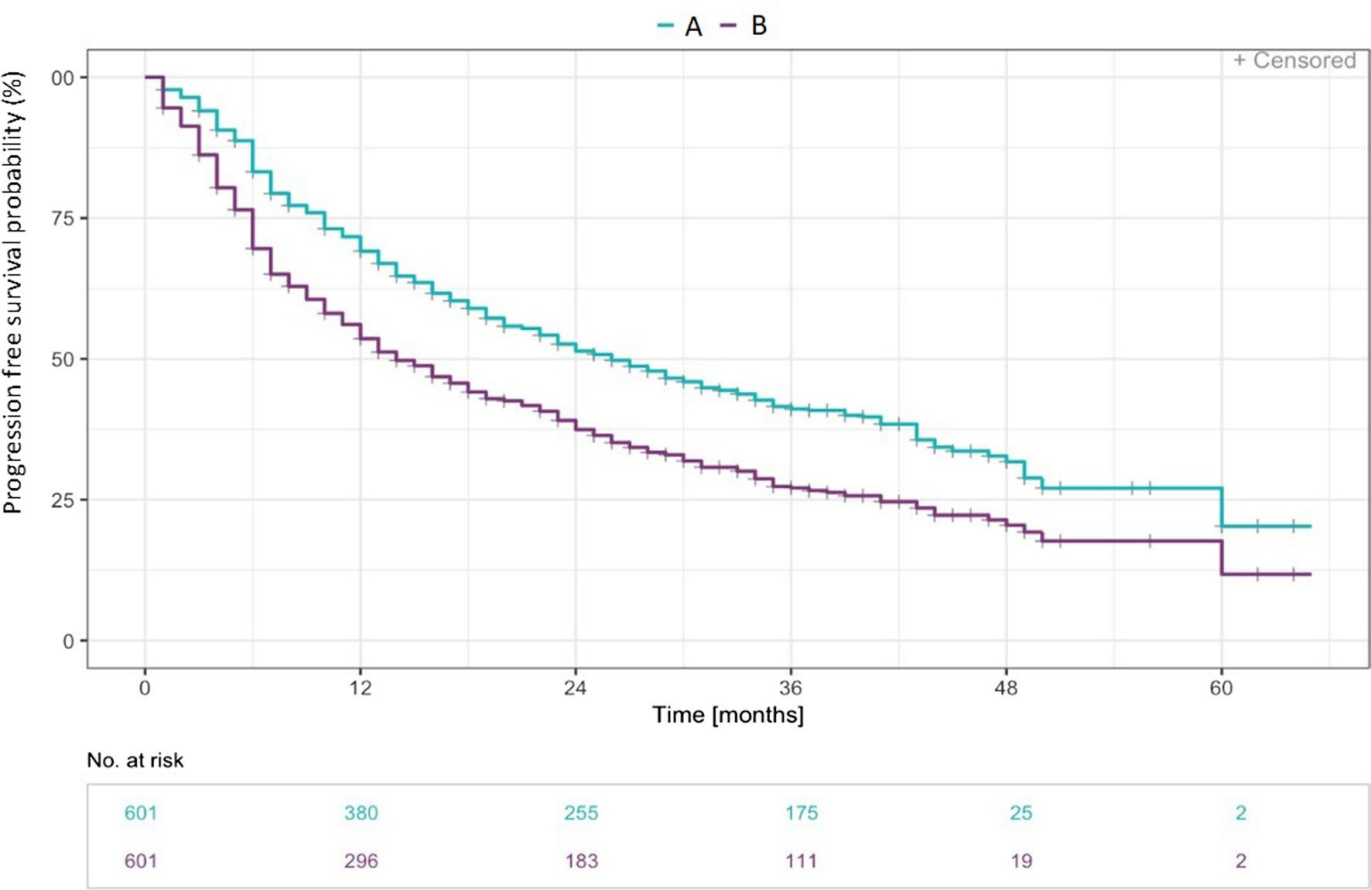
Time to ILD progression in months after definition A and B—Kaplan Meier curve. **A** ΔForced vital capacity (FVC) ≥ 10% or respiratory hospitalisation or death (n= 340); **B** ΔFVC ≥ 10% or all cause hospitalisation or death (n= 415)

The probability of progression free survival in association with a respiratory hospitalisation was 69.1% in the first 12 months and 51.4% in the second year compared to 53.6% within the first year and 37.4% in second for all cause hospitalisations (Fig. 2).

**Fig. 3:**
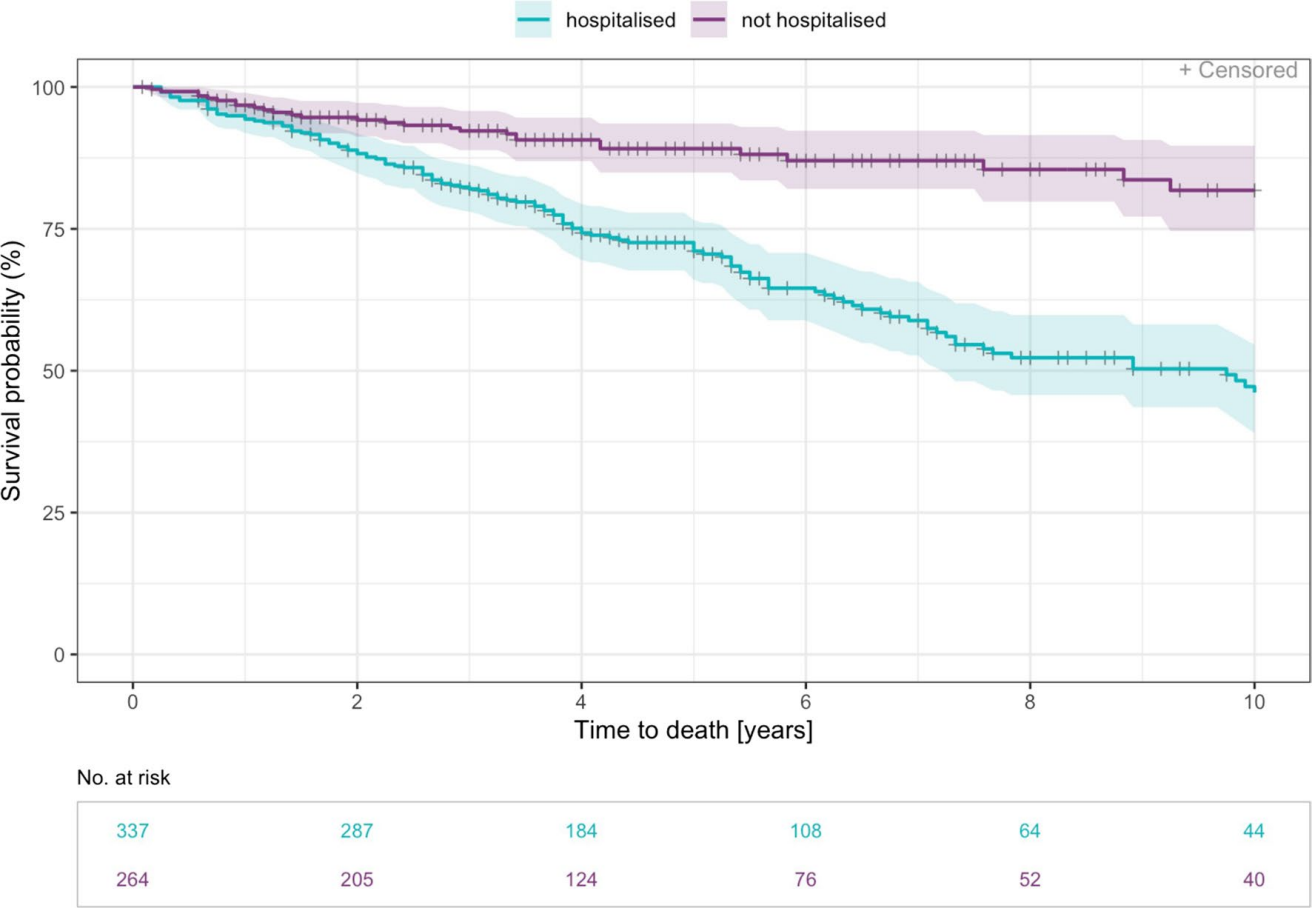
Time to death—Kaplan Meier curve for years 0–10 since diagnosis by all cause hospitalisation. Patients who have reported at least one hospitalisation that started after inclusion date are counted as hospitalised (n= 337). Median time to event was 9.7 years (range 0.2–34.4 years)

**Fig. 4:**
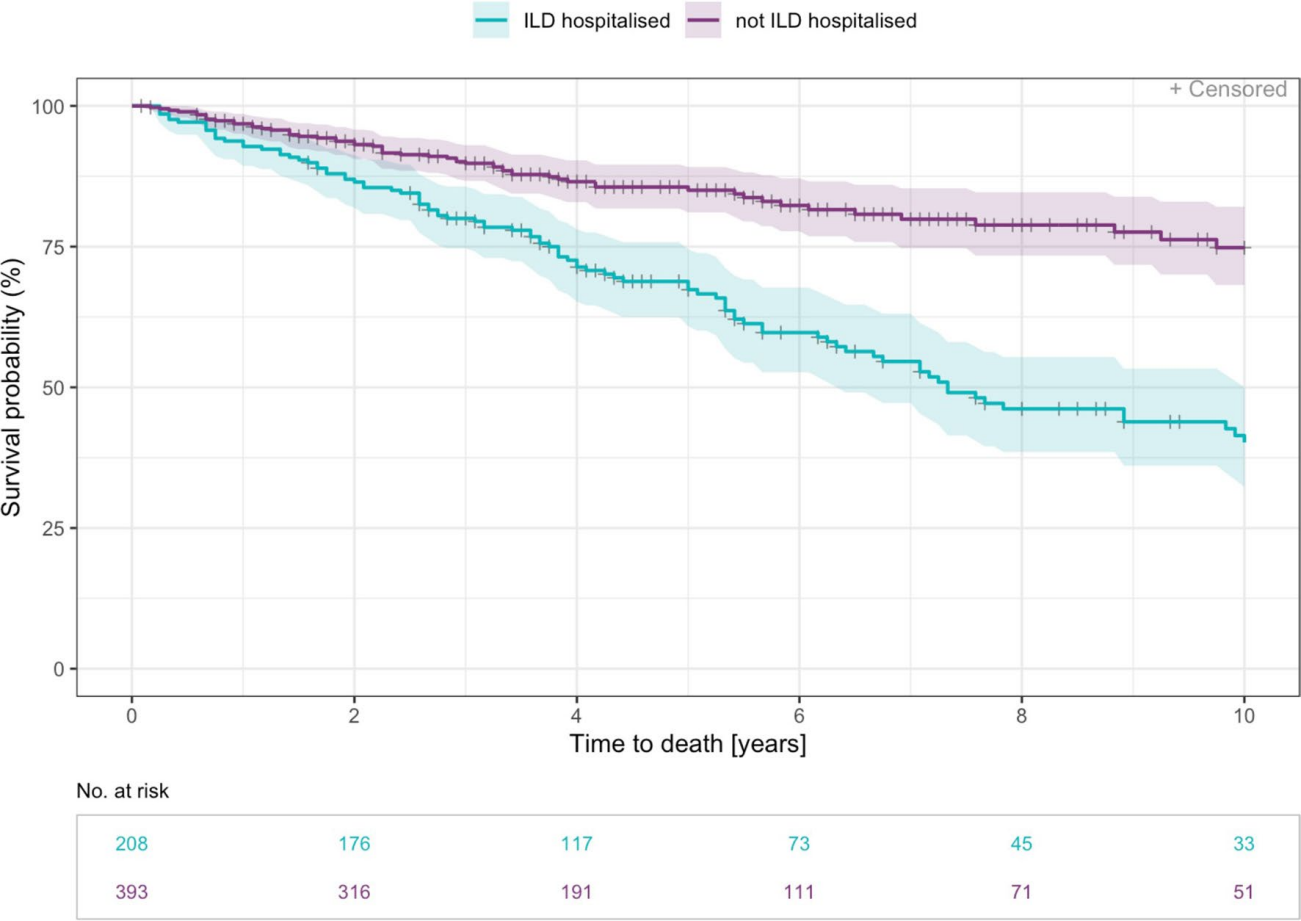
Time to death—Kaplan Meier curve for years 0–10 since diagnosis by ILD-hospitalisation. Non-elective ILD-hospitalisation from date of inclusion were considered (n= 208). Median time to event was 7.3 years (range 0.2–34.4 years)

**Fig. 5:**
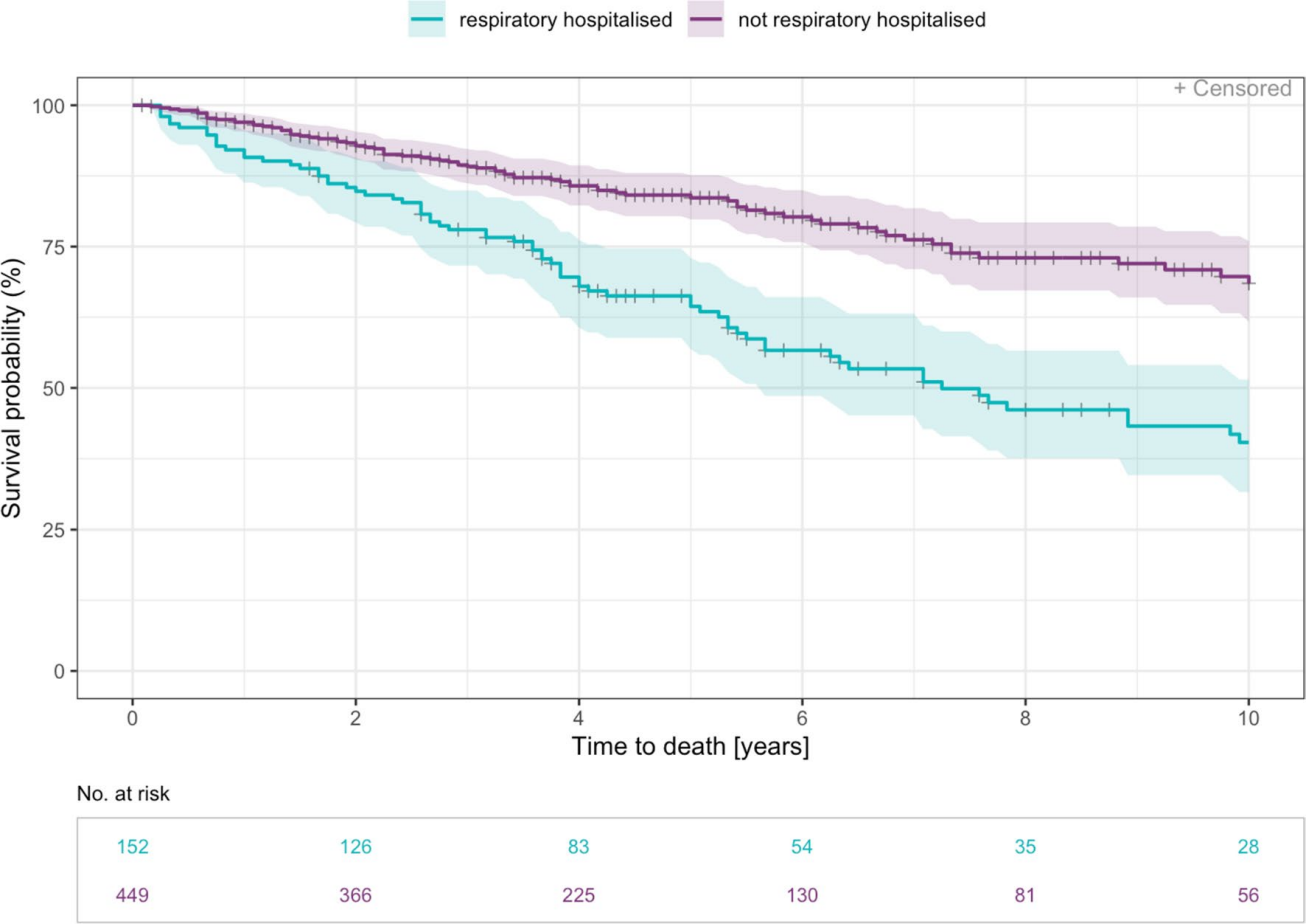
Time to death—Kaplan Meier curve for years 0–10 since diagnosis by respiratory hospitalisation. A respiratory hospitalisation was defined as exclusively non-elective caused by pneumonia, ILD exacerbation or pneumothorax (n= 152). Median time to event was 7.2 years (range 0.2–34.4 years)

Time to death was significantly increased in patients without all cause hospitalisations (HR 0.30 [0.20, 0.44], p < 0.001). The risk of death was also significantly decreased for patients without hospitalisations due to ILD (HR 0.36 [0.27, 0.49], p < 0.001) and without respiratory hospitalisations (HR 0.40 [0.30, 0.54], p < 0.001) (Figs. 3, 4 and 5).

### Factors influencing hospitalisations

Two analyses were performed to determine factors influencing hospitalisations. First, time to first hospitalisation was compared between different values of GAP index as well as for different ILD subtypes using cox regression models. Secondly, to analyse a wider range of possible influencing factors, multiple logistic regression was used to determine predictors of hospitalisation in general. The analyses using cox regression models showed that GAP index had a significant effect on time to first hospitalisation. In higher GAP stages II and III hospitalisations occurred earlier compared to GAP stage I, e.g. for GAP index III vs. I with a Hazard ratio of 2.99 (p < 0.001; Table3). Time to first hospitalisation was analysed in different ILD subtypes compared to IPF. Time to first hospitalisation as well as progression-free survival were lowest in connective tissue diseases with pulmonary involvement (CTD-ILD) with a median time to first hospitalisation of 9,8 months (range 0.5 to 53.45) as compared to 12.2 months (range 0.2 to 49.15) in IPF (p = 0.014). Significant effects on time to first hospitalisation and time to first ILD-hospitalisation were also found in granulomatous lung diseases compared to IPF (HR 0.35 and 0.23, p < 0.001; Table3). Kaplan Meier curves for time to first non-elective hospitalisation by ILD subtype are provided within the supplement (Additional file 1: Fig. S2).

**Table 3:** Output of Cox regression model of time to first hospitalisation and time to first ILD-hospitalisation analysis by GAP Index (n=601) and ILD subtype (n=468)

	Time to first hospitalisation		Time to first ILD-hospitalisation	
	HR [95% CI]	p value (Wald test)	HR [95% CI]	p value (Wald test)
GAP Index				
II vs. I	2.5 [1.91, 3.27]	< 0.001	2.36 [1.67, 3.33]	< 0.001
III vs. I	2.99 [2.24, 3.99]	< 0.001	3.13 [2.2, 4.47]	< 0.001
No PFT vs. I	2.14 [1.36, 3.36]	< 0.001	2.34 [1.35, 4.05]	0.002
ILD Subtype				
Granulomatous lung diseases vs. IPF	0.35 [0.25, 0.48]	< 0.001	0.23 [0.15, 0.36]	< 0.001
HP vs. IPF	0.82 [0.58, 1.18]	0.287	0.96 [0.64, 1.44]	0.844
CTD-ILD vs. IPF	1.59 [1.1, 2.3]	0.014	1.53 [0.99, 2.36]	0.055
Pneumoconiosis vs. IPF	0.68 [0.3, 1.54]	0.353	0.14 [0.02, 1.02]	0.052
Drug related ILD vs. IPF	0.97 [0.51, 1.85]	0.936	0.89 [0.41, 1.9]	0.758
Radiotherapy associated ILD vs. IPF	0.92 [0.34, 2.49]	0.873	0.34 [0.05, 2.44]	0.283
CPFE vs. IPF	1.29 [0.7, 2.38]	0.412	0.97 [0.43, 2.2]	0.936

For these analyses, Hazard Ratios were drawn from a comparison of the most relevant ILD subtypes and IPF. HR= Hazard Ratio, CI= confidence interval, No PFT= No pulmonary function testing, IPF= Idiopathic pulmonary fibrosis, HP= Hypersensitivity pneumonitis, CTD-ILD= Rheumatic and connective tissue diseases with pulmonary involvement, CPFE= Fibrosis in emphysema patients without signs of other ILDs

In the second step, multiple logistic regressions with forward selection were set to develop a model for the prediction of all cause, ILD-related and respiratory hospitalisations. In univariate analysis, baseline pulmonary function parameters showed an association both to all cause (FVC: OR = 0.98, p < 0.001; DLCO-SB: OR = 0.97, p < 0.001) and ILD-related hospitalisations (FVC: OR = 0.98, p < 0.001; DLCO-SB: OR = 0.96, p < 0.001). For all cause hospitalisations pulmonary hypertension (OR = 2.53, p = 0.005) and age (OR = 1.05, p < 0.001) were significant risk factors, while there was a non-significant association to reflux (OR = 1.23, p = 0.297), male sex (OR = 1.23, p = 0.217), concomitant emphysema (OR = 1.30, p = 0.323) and smoking (OR = 1.22, p = 0.220). ILD-related hospitalisations were also associated with pulmonary hypertension (OR = 2.84, p < 0.001).

In multiple regression models regarding ILD-related hospitalisations the combination of an emphysema in unclassifiable ILD (OR = 2.13, p = 0.001) and a current or ex-smoking status in other granulomatous ILD (OR = 3.08, p = 0.005) were identified as the most important risk factors. Also, DLCO-SB showed an association (OR = 1.00, p < 0.001; Additional file 1: Table S1). The strongest predictor for a respiratory hospitalisation was the combination of CTD-ILD and a concomitant emphysema (OR = 2.16, p = 0.006; Table4). An increased risk was also shown for male sex and HP of unknown origin (OR = 1.69, p = 0.008; Table4).

**Table 4:** Results of multiple logistic regression with forward selection to predict respiratory hospitalisation (n=601)

Predictor variable	Odds Ratio	Standarderror	95%-CI	p-value
VCmax:DLCO-SB	1.00	0.000	[0.000; 0.000]	< 0.001
Pulmonary hypertension [yes]:Age	1.00	0.001	[0.002; 0.007]	0.001
BMI:ILD/Vasc	1.02	0.007	[0.009; 0.036]	0.001
Concomitant emphysema:ILD/CTD	2.16	0.276	[0.229; 1.313]	0.006
Sex [male]:ILD/u-HP	1.69	0.196	[0.140; 0.908]	0.008

Logistic regression with forward selection was used to develop a model to predict respiratory hospitalisation (yes/no), relevant interactions are presented in this table. *Vasc* Vasculits, *u-HP* HP of unknown origin

## Discussion

In the present study we report a high rate of hospitalisations in patients with various ILDs with a large share of ILD-related hospitalisations. Notably, hospitalisations were associated with impaired prognosis. Besides elective diagnostic procedures relevant reasons for admission were acute ILD exacerbations and pulmonary infections. This is in line with a large German claims data study, which reported among 36.816 patients a hospitalisation rate of 71.2% for non-ILD-related and 56.6% for ILD-related reasons [18]**.** For IPF, Brown et al. and Cottin et al. reported an even higher rate of ILD-related hospitalisations compared to our data, potentially due to more frequent acute exacerbations in IPF than in other ILDs [8, 14].

To identify factors influencing hospitalisation, time to first hospitalisation was compared between different values of the GAP index and between different ILD subtypes. In addition, sex, age, BMI, pulmonary function parameters, ILD subtype, pulmonary hypertension, reflux, concomitant emphysema, and smoking behaviour were analysed in multiple logistic regression models to determine which of these variables are predictors of hospitalisation in general. Risk factors for hospitalisations included advanced disease, reflected by higher GAP stages associating with more frequent hospitalisations. Thus, the GAP Index, originally developed to predict prognosis in patients with IPF [17], was identified here to also predict hospitalisations in all ILDs. Baseline pulmonary function parameters were also associated with hospitalisations as described in SSc-ILD [13] or IPF [19], as well as in AE-IPF where disease severity as assessed by lung function associates with the probability of an acute exacerbation [20, 21].

Other predictors for non-elective hospitalisations were pulmonary hypertension and emphysema, the former in line with GAP stage most probably reflecting disease severity. The impact of emphysema is only poorly understood. Potential reasons might be a higher probability of pulmonary infections in a severely impaired lung due to both, ILD and emphysema; it is however unknown whether acute exacerbations of the co-existing COPD might also act as a co-factor.

Disease entity plays a decisive role in predicting hospitalisation with relevant differences e.g. between IPF and granulomatous lung diseases. This is in line with German claims data by Wälscher et al. reporting a lower frequency in non-ILD-related hospitalisations for sarcoidosis [18]. Possible reasons are younger age, less comorbidities and usually a relatively good prognosis in sarcoidosis and other granulomatous lung diseases [18]. A higher risk for hospitalisation and impaired survival was seen in rheumatic and connective tissue diseases with pulmonary involvement (CTD-ILD). These findings may partly be due to hospitalisations for the rheumatic manifestations, complications like pulmonary hypertension or therapy associated pulmonary infections related hospitalisations [22, 23].

The burden of hospitalisations was reflected by an association to survival. A study with almost 600 patients suffering from IPF showed that both all-cause and respiratory-related hospitalisations were strongly associated with mortality [8]. However, our data suggest an association beyond IPF for all ILDs. Presumably, non-elective hospitalisations appear mainly in progressive disease and thus represent a disease progression on its own [8, 18]. In line with this, the progression criteria reported here might represent future endpoints in clinical trials. However, possible regional differences with regard to the hospitalisation of patients with ILDs must be taken into account. Precisely because hospitalisations have such a major impact on mortality, approaches for prevention are warranted. Vaccinations could contribute in this respect. This was shown recently by an epidemiological claims data analysis for the vaccination against influenza in the sense of a reduced all-cause mortality in half of the seasons observed. However, Marijic et al. reported also an overall low vaccination rate in ILD patients [24]. Additionally, early detection and optimised treatment of comorbidities are essential.

Strengths of our analyses include the large number of patients recruited from different healthcare facilities as well as the prospective design. This enables to reflect the “real world”-situation of patients with ILDs and is therefore of high value. In particular, our results highlight the great impact of hospitalisations on mortality. Noteworthy, possible risk factors were identified, which could help to improve patient care.

However, also some limitations applied. Due to the study character as observational and nonrandomised, no causal associations may be derived. In addition, both prevalent and incident patients were included. The diagnoses were mostly, but not exclusively, made on an interdisciplinary basis. CT imaging was not considered in the registry. Additionally, clinical decisions of the physicians may differ, especially because patients were treated in centres with different levels of expertise in ILD [16]. So, potentially allocation or channelling bias could be caused and confound the association between treatment and outcomes [16]. The higher number of admissions in the first year of the registry compared to the following years might be caused by a decreasing number of patients in the registry over the years. Therefore, this point should be interpreted with caution. Nevertheless, it illustrates the high burden of disease in interstitial lung diseases. As only pulmonary hypertension, emphysema and GERD were recorded as comorbidities, no statements about cardiac or other comorbidities can be made.

## Conclusion

In the EXCITING-ILD registry hospitalisations were frequent and occurred mainly early. Non-elective hospitalisations are strongly correlated with prognosis and thus may serve as a surrogate endpoint in clinical trials. Our results represent a crucial contribution in understanding predisposing factors for hospitalisation in ILD and its major impact on mortality. Further studies to characterize the most vulnerable patient group as well as approaches to prevent hospitalisations such as vaccinations are warranted.

### Supplementary Information

**Additional file 1: Figure S1**. Time to first ILD-hospitalisation in months—Kaplan Meier curve Non-elective ILD-hospitalisation from date of inclusion were considered (n=208). **Figure S2**. Time to first non-elective hospitalisation in months by ILD subtypes—Kaplan Meier curve. Time to first non-elective hospitalisation was defined as the first non-elective hospitalisation from inclusion to the registry (n=322). The most relevant ILD subtypes were considered. *IPF* Idiopathic pulmonary fibrosis, *NSIP* non-specific interstitial pneumonia, *COP* cryptogenic organizing pneumonia, *uILD* not classifiable IIP, *Sarc* sarcoidosis, *CTD* rheumatic and connective tissue diseases with pulmonary involvement, *RA* rheumatoid arthritis-associated ILD, *LAM* pulmonary lymphangioleiomyomatosis, *PLCH* pulmonary langerhans´ cell histiocytosis, *PAP* pulmonary alveolar proteinosis, *EP* eosinophilic pneumonia. **Table S1**. Results of multiple logistic regression with forward selection to predict ILD hospitalisation (n=601). Logistic regression with forward selection was used to develop a model to predict ILD hospitalisation (yes/no), relevant interactions are presented in this table. *DLCO-SB* diffusing capacity for carbon monoxide (CO) – single breath, *ILD* interstitial lung disease, *u-HP* HP of unknown origin, *IPF* idiopathic pulmonary fibrosis, *CTD* connective tissue disease, *Vasc* Vasculits, *uILD* not classifiable idiopathic interstitial pneumonia, *NSIP* non-specific interstitial pneumonia, other GRAN-ILD: other e.g. involvement in chronic inflammatory liver and gut diseases, except hypersensitivity pneumonitis [EAA].

## Data Availability

The datasets used and/or analysed during the current study are available from the corresponding author on reasonable request.

## Abbreviations

BMI: Body mass index
CI: Confidence interval
COP: Cryptogenic organizing pneumonia
CPFE: Fibrosis in emphysema patients without signs of other ILDS
CTD: Connective tissue disease
DLCO-SB: Diffusing capacity for carbon monoxide (CO) single breath
EP: Eosinophilic pneumonia
EXCITING-ILD: Exploring Clinical and Epidemiological Characteristics of Interstitial Lung Diseases
FAS: Full analysis set
FEV1: Forced expiratory volume in 1s
FVC: Forced vital capacity
HP: Hypersensitivity pneumonitis
HR: Hazard ratios
ILD: Interstitial lung disease
IPF: Idiopathic pulmonary fibrosis
LAM: Pulmonary lymphangioleiomyomatosis
NIV: Non-invasive ventilation
NSIP: Non-specific interstitial pneumonia
OR: Odd’s ratio
PAP: Pulmonary alveolar proteinosis
PFT: Pulmonary function testing
PLCH: Pulmonary langerhans´ cell histiocytosis
RA: Rheumatoid arthritis
SD: Standard deviation
SSc: Systemic sclerosis
u-HP: HP of unknown origin
uILD: Not classifiable IIP
Vasc: Vasculits
