# Supplementary material for: Hospitalisation patterns in interstitial lung diseases: data from the EXCITING-ILD registry
Source: Respir Res. 2024 Jan 4;25:5. doi: 10.1186/s12931-023-02588-y (PMC10765927; doi:10.1186/s12931-023-02588-y)
Supplement: Supplementary file 1 — Additional file 1: Figure S1. Time to first ILD-hospitalisation in months—Kaplan Meier curve Non-elective ILD-hospitalisation from date of inclusion were considered (n=208). Figure S2. Time to first non-elective hospitalisation in months by ILD subtypes—Kaplan Meier curve. Time to first non-elective hospitalisation was defined as the first non-elective hospitalisation from inclusion to the registry (n=322). The most relevant ILD subtypes were considered. IPF Idiopathic pulmonary fibrosis, NSIP non-specific interstitial pneumonia, COP cryptogenic organizing pneumonia, uILD not classifiable IIP, Sarc sarcoidosis, CTD rheumatic and connective tissue diseases with pulmonary involvement, RA rheumatoid arthritis-associated ILD, LAM pulmonary lymphangioleiomyomatosis, PLCH pulmonary langerhans´ cell histiocytosis, PAP pulmonary alveolar proteinosis, EP eosinophilic pneumonia. Table S1. Results of multiple logistic regression with forward selection to predict ILD hospitalisation (n=601). Logistic regression with forward selection was used to develop a model to predict ILD hospitalisation (yes/no), relevant interactions are presented in this table. DLCO-SB diffusing capacity for carbon monoxide (CO) – single breath, ILD interstitial lung disease, u-HP HP of unknown origin, IPF idiopathic pulmonary fibrosis, CTD connective tissue disease, Vasc Vasculits, uILD not classifiable idiopathic interstitial pneumonia, NSIP non-specific interstitial pneumonia, other GRAN-ILD: other e.g. involvement in chronic inflammatory liver and gut diseases, except hypersensitivity pneumonitis [EAA]. [file 12931_2023_2588_MOESM1_ESM.docx]

**Additional file 1**


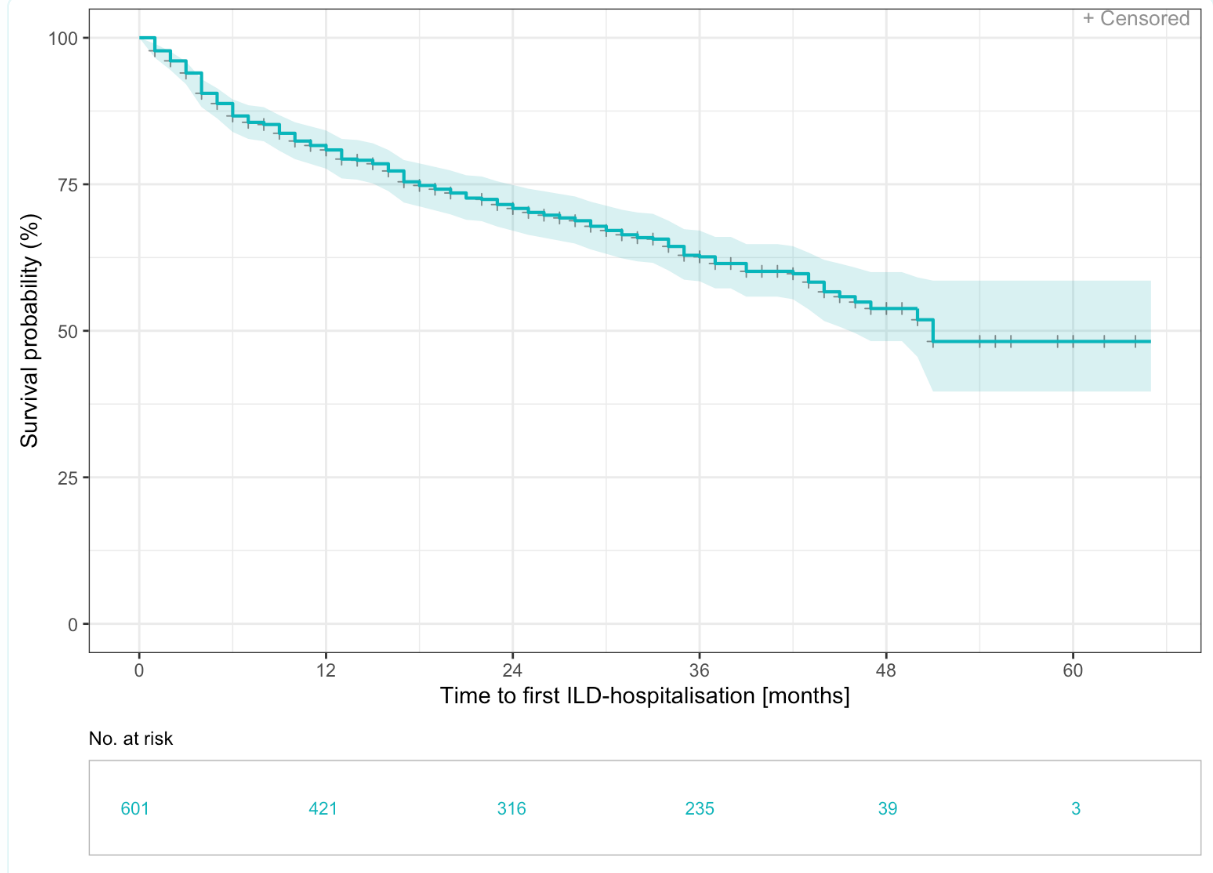


Event free probability (%)

**Figure S1** Time to first ILD-hospitalisation in months - Kaplan Meier curve

Non-elective ILD-hospitalisation from date of inclusion were considered (n= 208).


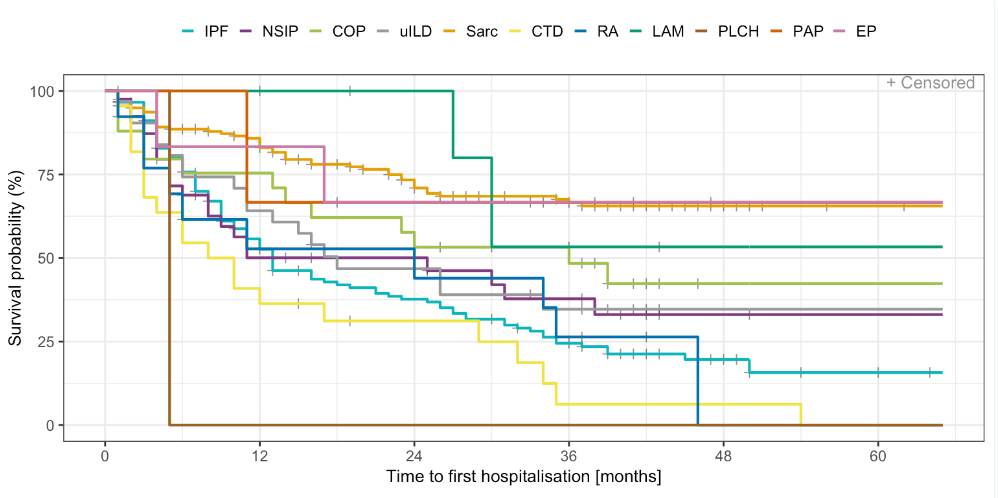


Event free probability (%)


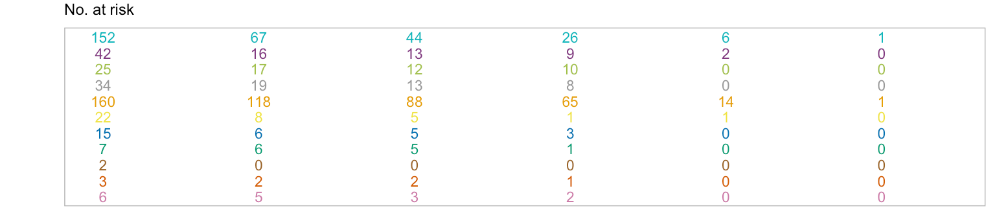


**Figure S2** Time to first non-elective hospitalisation in months by ILD subtypes - Kaplan Meier curve

Time to first non-elective hospitalisation was defined as the first non-elective hospitalisation from inclusion to the registry (n= 322). The most relevant ILD subtypes were considered. IPF= Idiopathic pulmonary fibrosis, NSIP= non-specific interstitial pneumonia, COP= cryptogenic organizing pneumonia, uILD= not classifiable IIP, Sarc= sarcoidosis, CTD= rheumatic and connective tissue diseases with pulmonary involvement, RA= rheumatoid arthritis-associated ILD, LAM= pulmonary lymphangioleiomyomatosis, PLCH= pulmonary langerhans´ cell histiocytosis, PAP= pulmonary alveolar proteinosis, EP= eosinophilic pneumonia.

| **Predictor variable** | **Odds Ratio** | **Standarderror** | **95%-CI** | **p-value** |
| --- | --- | --- | --- | --- |
| DLCo-SB | 0.995 | 0.001 | [-0.007; 0.003] | <0.001 |
| Age:ILD/u-HP | 1.006 | 0.002 | [0.003; 0.009] | <0.001 |
| Pulmonary hypertension [Yes]:Age | 1.004 | 0.001 | [0.001; 0.006] | 0.006 |
| ILD/IPF:Smoker [Yes] | 1.349 | 0.066 | [0.169; 0.430] | <0.001 |
| Sex [Male]:ILD/CTD | 1.747 | 0.145 | [0.273; 0.843] | 0.001 |
| BMI:ILD/Vasc | 1.024 | 0.007 | [0.010; 0.038] | 0.001 |
| Concomitant emphysema [Yes]:ILD/uILD | 2.133 | 0.232 | [0.302; 1.213] | 0.001 |
| Reflux [Yes]:ILD/NSIP | 1.549 | 0.144 | [0.154; 0.721] | 0.003 |
| Vcmax:Smoker [Yes] | 0.998 | 0.001 | [-0.003; 0.001] | 0.004 |
| ILD/Other GRAN-ILD:Smoker [Yes] | 3.082 | 0.401 | [0.339;1.912] | 0.005 |

**Table S1** Results of multiple logistic regression with forward selection to predict ILD hospitalisation (n= 601). Logistic regression with forward selection was used to develop a model to predict ILD hospitalisation (yes/no), relevant interactions are presented in this table. DLCO-SB: diffusing capacity for carbon monoxide (CO) – single breath, ILD: interstitial lung disease, u-HP: HP of unknown origin, IPF: idiopathic pulmonary fibrosis, CTD: connective tissue disease, Vasc: Vasculits, uILD: not classifiable idiopathic interstitial pneumonia, NSIP: non-specific interstitial pneumonia, other GRAN-ILD: other e.g. involvement in chronic inflammatory liver and gut diseases, except hypersensitivity pneumonitis [EAA].
